# Central cholinergic synapse formation in optimized primary septal-hippocampal co-cultures

**DOI:** 10.1007/s10571-020-00948-6

**Published:** 2020-08-28

**Authors:** Sarra Djemil, Claire R. Ressel, Mai Abdel-Ghani, Amanda K Schneeweis, Daniel T.S. Pak

**Affiliations:** 1 Department of Pharmacology and Physiology, Georgetown University Medical Center, Washington, District of Columbia, USA; 2 Department of Biology, Georgetown University, Washington, District of Columbia, USA; 3 Interdisciplinary Program in Neuroscience, Georgetown University Medical Center, Washington, District of Columbia, USA

**Keywords:** Septal, Cholinergic, septal-hippocampal co-culture, basal forebrain cholinergic neurons, cholinergic synapse, primary culture

## Abstract

Septal innervation of basal forebrain cholinergic neurons to the hippocampus is critical for normal learning and memory and is severely degenerated in Alzheimer’s disease. To understand the molecular events underlying physiological cholinergic synaptogenesis and remodeling, as well as pathological loss, we developed an optimized primary septal-hippocampal co-culture system. Hippocampal and septal tissue were harvested from embryonic Sprague-Dawley rat brain and cultured together at varying densities, cell ratios, and in the presence of different growth factors. We identified conditions that produced robust septal-hippocampal synapse formation. We used confocal microscopy with primary antibodies and fluorescent ligands to validate that this system was capable of generating developmentally mature cholinergic synapses. Such synapses were comprised of physiological synaptic partners and mimicked the molecular composition of *in vivo* counterparts. This co-culture system will facilitate the study of the formation, plasticity, and dysfunction of central mammalian cholinergic synapses.

## INTRODUCTION

Basal forebrain cholinergic neurons (BFCNs) are essential for proper memory formation within the hippocampus (Coyle et al. 1983; Luchicchi et al. 2014; Ballinger et al. 2016; Hampel et al. 2017). A great deal of information has accrued regarding plasticity of hippocampal neurons, aided by the availability of high quality primary hippocampal culture preparations that have faithfully reproduced many *in vivo* characteristics and have been extensively and productively used for decades since their initial introduction (Banker and Cowan 1977). However, far less detail is known about the plasticity of mammalian central nervous system (CNS) cholinergic synapse. Importantly, BFCNs are among the first neurons that succumb to degeneration in Alzheimer’s disease (AD), but the mechanisms underlying the cell-type specific vulnerability of these neurons in AD remain unknown. Molecular studies examining these and other neuropathological issues have been hampered, in part by the lack of a robust cultured neuron model of BFCNs.

Septal cultures originally derived from fetal rats, pioneered by early work from Hartikka and Hefti, provided a major breakthrough by identifying the dissection margins of the developing septal area, which includes the diagonal brand of Broca, and revealing the reliance of cholinergic neuron survival on plating density and the presence of nerve growth factor (NGF) (Hartikka and Hefti 1988). Subsequently, hippocampal membranes were found to offer trophic support that is independent of NGF (Emerit et al. 1989), leading to interest in co-culturing septal and hippocampal neurons. Nevertheless, such co-cultures have not gained widespread usage. Several hurdles faced when replicating these early studies included the complexity of the media composition and relative dearth of technical details on culture maintenance. There was also uncertainty regarding the true representation of cholinergic neurons in the Hartikka and Hefti study, which attempted to identify these neurons using acetylcholinesterase (AChE) staining. However, this enzyme is present in both cholinergic and cholinoceptive neurons (Andrä et al. 1988; Zoli 2000), and therefore the use of AChE as a marker would greatly overestimate the BFCN count.

In the rodent basal forebrain, cholinergic neurons represent only ~5% of the total neuronal cell population (Emerit et al. 1989; Gritti et al. 2006). The fraction of the BFCNs that survive under culture conditions is dependent on a multitude of factors, including the presence of neurotrophic factors, serum, and appropriate target neurons. Therefore, we sought to identify the optimal culturing conditions that would support mature cholinergic neuron survival and morphology similar to the intact rodent brain, ensure the robust survival and integrity of hippocampal target neurons, and enable the formation of bona fide central cholinergic synapses.

## MATERIALS AND METHODS

### Animals

All experimental procedures using animals were approved and performed in accordance with regulations of the Georgetown University Institutional Animal Care and Use Committee (IACUC protocol #15-026-100232). A total of 15 pregnant Sprague-Dawley rat mothers (8–10-week-old females, RRID: RGD_734476) were obtained from Charles River (Raleigh, NC) and used for these studies, and each analysis included independent cultures from at least three animals. This study was not pre-registered as it does not include any human subject data.

### Cell culture

Timed-pregnant Sprague-Dawley dams were singly housed for 2 days in individually ventilated cages, with *ad libitum* access to food and water. At embryonic day 14 or 19 (E14 or 19), pregnant rats were euthanized using a flow-regulated carbon dioxide chamber to minimize asphyxiation distress, and death was verified by toe pinch and decapitation. Anesthetics were not used due to the possible interference with proper neuronal growth in culture (Mintz et al. 2012). Immediately following euthanasia, embryos were removed by laparotomy. Septal and hippocampal tissues were separately harvested according to a procedure modified from previous published protocols, including cultured striatal cholinergic interneuron preparation (Hartikka and Hefti 1988; Pak et al. 2001; Schnitzler et al. 2008; Schock et al. 2010; Haam et al. 2018). Briefly, brain tissue was digested for 15 minutes with 0.1% trypsin and by mechanical trituration, followed by the application of trypsin inhibitor (Sigma, cat. # T7659) to terminate the trypsin reaction. A total of 150,000 cells were seeded into 12-well culture plates at an approximate density of 300 cells/mm^2^ using different ratios of septal:hippocampal cells (3:1 (112,500:37,500 cells), 2:1 (100,000:50,000 cells), or 1:1 (75,000:75,000 cells)). A separate condition tested a ratio of 7.5:1 (75,000:10,000 cells). Cells were plated onto acid-washed borosilicate cover glasses coated with poly-D-lysine (Sigma, cat. # P0899) and laminin (Sigma, cat. # L2020). Septal-hippocampal co-cultures were continuously maintained in Neurobasal medium (Gibco, cat. # 2103–049) with SM1 supplement (StemCell, cat. # 05711), 0.5 mM glutamine, 12.5 μM glutamate and 100 ng/mL 2.5S NGF (Sigma, cat. # N6009) and stored in a 5% CO_2_ and 95% O_2_ humidified incubator at 37°C. In addition to NGF, several agents were tested to determine the optimal growth of septal-hippocampal cultures but were ultimately not chosen in our final culture protocol (see Supplemental Materials): bone morphogenetic protein 9 (BMP9, 10 ng/mL) (R&D systems, cat. # 3209-BP-010), basic fibroblast growth factor (bFGF, 20 ng/mL) (PeproTech, cat. # 100–18B), a cocktail of all three growth factors (3xGF), and nicotine (500 nM) (Millipore Sigma, cat. # N5260). Half of the media was replaced with fresh media lacking glutamate on days *in vitro* (DIV) 5 and 14. Experiments were performed on cultures at DIV 21.

### Antibodies and Fluorescent Ligands

Rabbit anti-vesicular acetylcholine transporter (VAChT) (1:500, cat. # 139 103, Synaptic Systems, RRID: AB_887864), guinea pig anti-VAChT (1:500, cat. # 139 105, Synaptic Systems, RRID: AB_1089397), mouse anti-CHT1 (1:500, cat. # 216 011, Synaptic Systems RRID: AB_2301977), goat anti-ChAT (1:400, cat. # AB144p, Millipore, AB_2079751), rabbit anti-GAD65 (1:250, cat. # AB5082, Millipore, RRID: AB_2107925), mouse anti-VGAT (1:500, cat. # 131 011, Synaptic Systems, RRID: AB_887872), rabbit anti-gephyrin (1:500, cat. # 147 008, Synaptic Systems, RRID: AB_2619834), mouse anti-PSD-93 / Chapsyn-110 (1:250, cat. # 75–284, UC Davis/NIH NeuroMab, RRID: AB_11001825), rabbit anti-PSD95 (1:200, cat. # 3450, Cell Signaling Technology, RRID: AB_2292883), chicken anti-MAP2 (1:1000, cat. # AB15452, Millipore Sigma, RRID: AB_805385), donkey anti-mouse Alexa Fluor 555 (1:300, cat. # A-31570, Thermo Fisher Scientific, RRID: AB_2536180), donkey anti-rabbit Alexa Fluor 555 (1:300, cat. # A-31572, Thermo Fisher Scientific, RRID: AB_162543), donkey anti-goat Alexa Fluor 555 (1:300, cat. # A-21432, Thermo Fisher Scientific, RRID: AB_2535853), donkey anti-rabbit Alexa Fluor 647 (1:300, cat. # A-31573, Thermo Fisher Scientific, RRID: AB_2536183), donkey anti-mouse Alexa Fluor 647 (1:300, cat. # A-31571, Thermo Fisher Scientific, RRID: AB_162542), donkey anti-rabbit Alexa Fluor 488 (1:300, cat. # A-21206, Thermo Fisher Scientific, RRID: AB_2535792), goat anti-guinea pig Alexa Fluor 488 (1:300, cat. # A-11073, Thermo Fisher Scientific, RRID: AB_2534117), goat anti-chicken Alexa Fluor 647 (1:250, cat. # A21449, ThermoFisher, RRID: AB_1500594), and α-Bungarotoxin (α-BTX) Alexa Fluor 555 (1 μg/mL, cat. # AB35451, Thermo Fisher Scientific, RRID: AB_2617152). For validation, see Online Resource Table 1.

### Live Fluorescent Ligand-binding

To ensure specificity of the fluorescent ligand α-BTX Alexa Fluor 555, a competition assay was performed using the α7 nAChR antagonist, methyllycaconitine (MLA) (Tocris Biosciences, cat. # 1029). Several cultures from independent animals were pretreated for 30 minutes with MLA in a 5% CO_2_ and 95% O_2_ humidified incubator at 37°C. Following this incubation period, under darkened conditions (using low level indirect lamp light) 1 μg/mL α-BTX Alexa Fluor 555 was fed directly into culture wells with and without MLA, and incubated for 15-minutes. Cultures were then gently washed 3 times for 2 minutes each using prewarmed Neurobasal media (Gibco cat. # 2110349) (equilibrated in 12-well culture plates in the incubator). Immediately following the media washes, the coverglass was then dipped 3 times for 30 seconds each into sterile prewarmed phosphate-buffered saline (PBS) (Gibco cat. # 10010023) (equilibrated in 12-well culture plates in the incubator). Cover glasses containing cultured neurons were then immediately fixed and used for immunocytochemistry in a dark chamber following the procedures described below.

### Immunocytochemistry

For immunocytochemistry, primary cultured septal-hippocampal neurons (DIV 21) were fixed in 1% paraformaldehyde (PFA)/4% sucrose for 7 minutes, followed by methanol (−20°C) for 7 minutes. Neurons were washed 3 times, 5 minutes each in sterile PBS and then incubated overnight at 4°C with primary antibodies in GDB buffer (30 mM phosphate buffer, pH 7.4, containing 0.1% gelatin, 0.3% Triton X-100, 450 mM NaCl). Neurons were washed 3 times for 15 minutes each in PBS prior to adding secondary antibodies (Alexa 647-, Alexa 555- and Alexa 488-conjugated, ThermoFisher) in GDB for two hours at 25°C. Neurons were again washed 3 times for 15 minutes each in PBS then mounted using VectaShield (Vector Laboratories, cat. # H-1000).

### Percent of Cholinergic Neurons in Culture

We defined cholinergic neurons as cells with strong VAChT expression within the cell body. We employed VAChT as our primary cholinergic marker due to its greater specificity in revealing cholinergic networks (Wong et al. 1999) compared to AChE (Hartikka and Hefti 1988) and its ability to visualize cholinergic presynaptic terminals. For VAChT-positive neuron counts, images were acquired using an Axiovert 200M microscope (Zeiss) for epifluorescence. The experimenter was blinded to culture condition while acquiring and analyzing images. Random fields with approximately 25% coverage of the coverslip surface area were selected to count cells. The percent of cholinergic neurons present per group was calculated as follows: average number of VAChT+ (cholinergic) neurons divided by the average number of MAP2-positive cells (all neurons) x 100.

### Confocal Imaging

To identify puncta for synaptic markers, images were acquired using a Leica SP8 confocal microscope and Leica Application Suite X (LAS X) software (Leica Microsystems). Sequential scan mode (2 sequences for double- and 3 sequences for triple-staining) was used to minimize crosstalk. Emission filter wavelengths were constrained to avoid back bleed-through. All imaging was held at constant parameters across groups to allow for comparisons.

### α-BTX Intensity

Background was quantified using the *Measure* function, then subtracted using the *Math/Subtract* function in FIJI software (Public Domain). Images were thresholded at a constant value (2x background), resulting in the selection of regions with intense staining, which were primarily soma and puncta. Regions of interest were selected manually using the *ROI Manager* in FIJI, leading to the selection of puncta and the immediately adjacent dendrite region (identified by MAP2 staining). Integrated intensity was obtained using the *Measure* function within FIJI. The intensity of each ROI was measured from original images (no brightness or contrast adjustments) from at least 3 independent culture preparations and averaged to minimize measuring variability.

### Distance Quantification

#### Soma size.

Confocal images were calibrated using the embedded scale bar and *Set-Scale* function in FIJI. Soma size of VAChT+ or PSD-93+ neurons was captured using the *Straight-Line* tool along the longest axis within the soma and recorded using the *Measure* function.

#### Between puncta.

Cholinergic innervation was defined as segments in which VAChT puncta colocalized with target dendrites (MAP2 positive neurites emanating from VAChT- cell bodies) along contiguous linear stretches of at least 5 μm in length (Takács et al. 2018). For analysis of distance between VAChT puncta and postsynaptic markers (αBTX, PSD-93, and gephyrin), measurements were made exclusively along these defined innervated segments. Distances of all VAChT puncta less than 5 μm away from the nearest postsynaptic puncta were then measured and their frequency distribution displayed as percentages.

### Colocalization

Colocalization along VAChT-innervated segments is reported as the index of correlation I_corr_. The index represents fraction of positively correlated (colocalized) pixels (0–1) in the analyzed images allowing for a highly sensitive quantitative measurement of colocalization. I_corr_ was determined using the *Colocalization Colormap* plugin in FIJI (Ayadi et al.; Jaskolski et al. 2005; Gorlewicz et al. 2020) according to the equation below, in which the calculated normalized mean deviation product (nMDP) represents the mathematical correlation between the intensities of corresponding green (VAChT) and red (synaptic marker) pixels (values range from −1 to 1):

$$\text{nNDP}=\frac{\left(\text{Ai}-\text{Av})(\text{Bi}-\text{Bv}\right)}{\left(\text{Amax}-\text{Av})(\text{Bmax}-\text{Bv}\right)}$$

Ai - intensity for the given pixel in the image A

Av - average intensity of the image A

Amax - maximum intensity of the image A

Bi - intensity for the given pixel in the image A

Bv - average intensity of the image B

Bmax - maximum intensity of the image B

### Software

For a complete list of software used in this work, see Online Resource Table 2.

### Statistics

Experimenters were blinded to the culture conditions during experimentation and data analysis. All data are presented as mean ± SEM (error bars) and individual experimental points are depicted in column or bar graphs. Statistical significance was set at p<0.05. For two-sample comparisons vs. controls, unpaired two-tailed Student’s t-test was used. Where analysis of variance (ANOVA) is carried out, the assumptions for normality (Shapiro-Wilk) and equality of variances (Bartlett’s test) are met. All data collected followed normal distributions; therefore, only parametric tests were used. Details for statistical and *post hoc* tests used and n number definition are provided within figure legends. No randomization was carried out for any of the experiments described here. Data summaries and statistical analysis were carried out using Graphpad Prism 8.

## RESULTS

### Characterization of septal cholinergic neurons *in vitro*

**Fig. 1:**
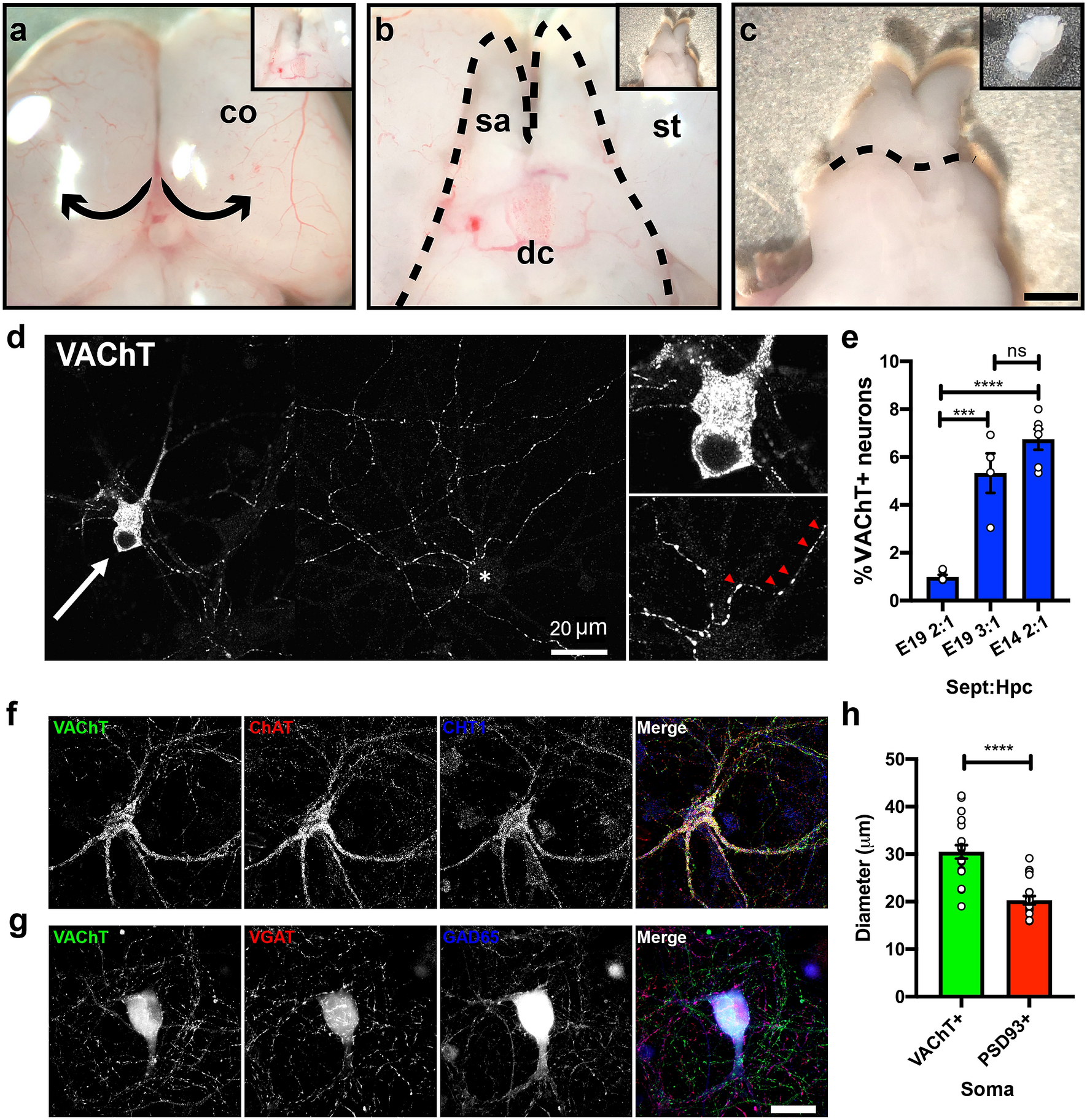
Septal dissection and characterization of cholinergic neurons within septal-hippocampal co-cultures. (**A-C**) Sprague Dawley embryonic rat brain dissection of septal region. Insets represent the result of the manipulation shown in the main image. (**a**) Exposing the septum by opening the cerebral hemispheres. (**b**) Dissection of the septal region along dotted outline. (**c**) Dissection of the septum by cutting along dashed line. Scale bar, 1 mm. Abbreviations: co, cortex; dc, diencephalon; sa, septal area; st, striatum. (**d**) Representative example of a VAChT-expressing cholinergic neuron strongly expressing VAChT (arrow) elaborating axonal arbors across a field of VAChT-lacking neurons, with apparent innervation of target cells (asterisk). Upper inset, higher magnification of cholinergic cell body. Lower inset, higher magnification of VAChT+ terminals innervating a target dendrite (red arrowheads). (**e**) Proportion of VAChT+ neurons in the culture as a percentage of total MAP2-positive cells (mean = 4273, n = 5 coverslips from 3 independent cultures) as a function of embryonic day at dissection and ratio of septal:hippocampal cells. E19 2:1 (1% VAChT+ cells = 42, n = 4 coverslips from 3 independent cultures); E19 3:1 (5.3% VAChT+ cells = 228, n = 4 coverslips from 3 independent cultures); E14 2:1 (6.7% VAChT+ cells = 288, n = 6 coverslips from 2 independent cultures). ***p<0.0005, ****p<0.0001, ns, not significant, one-way ANOVA with Tukey *post-hoc* test. (**f**) Immunocytochemistry of neurons from septal-hippocampal cultures using rabbit anti-VAChT (green), goat anti-ChAT (red), and mouse anti-CHT1 (blue) antibodies. All VAChT-expressing neurons were also positive for ChAT and CHT1 (12/12 neurons from n=3 coverslips obtained from 2 independent cultures). (**g**) Immunocytochemistry of neurons from septal-hippocampal cultures using a guinea pig anti-VAChT (green), mouse anti-VGAT (red), and rabbit anti-GAD65 (blue) antibodies. All VAChT-expressing neurons were also positive for VGAT and GAD65 (28/28 neurons from n=3 coverslips and 3 independent cultures). (**h**) Soma size calculated from 20 VAChT+ neurons (rabbit anti-VAChT) from n=4 coverslips and 5 independent cultures, and 20 PSD-93+ (and VAChT-) neurons from n=3 coverslips and 3 independent cultures; ****p<0.0001, unpaired two-tailed Student’s t-test.

Septal regions were dissected from fetal rats of embryonic age E14 or 19. The brain was extracted from the skull and placed on the ventral surface under a stereomicroscope in a petri dish containing Hanks Buffered Saline Solution. Using microscissors or fine forceps, the corpus callosum was cut to allow retraction of the cortical hemispheres (co) from the brain’s midline (Fig. 1a). Approximately a millimeter lateral to the midline, the septal area (sa) was exposed, which at this embryonic stage consists of a pair of conical structures (Fig. 1b). The adjacent tissue was excised and saved to perform hippocampal dissection, leaving the septal area and the diencephalon (dc). Using microscissors, the septal area was isolated by making a transverse cut along the borderline separating the septal area from the diencephalon (Fig. 1c). The hippocampi from the forebrain hemispheres were dissected according to previously published procedures (Lee et al. 2017).

Septal and hippocampal cells were co-cultured in an initial screen of 24 different conditions to test the effect of embryonic stage, cell-type density and ratio, and growth media on cholinergic neuron survival (Online Resource 1a). In line with previous reports (Hartikka and Hefti 1988; Kojima et al. 1994; Schnitzler et al. 2008), we observed that when compared to the absence of growth factor ((−) GF) condition, NGF is necessary for trophic support and survival of cholinergic neurons (Online Resource 1a). In addition, we tested basic fibroblast growth factor (bFGF), which was used in the growth of young (DIV 3) septal cultures (Schnitzler et al. 2008), and bone morphogenetic protein 9 (BMP9), due to its ability to enhance the expression of cholinergic markers VAChT, ChAT and CHT1 (Lopez-Coviella et al. 2005). Finally, nicotine is a cognitive enhancer that may be protective against AD (Newhouse et al. 1988), a hallmark of which is the degeneration of BFCNs. Nicotine also protects dopaminergic neurons from degeneration in Parkinson’s disease as well as *in vitro* (Baron 1986; Morens et al. 1995; Ryan et al. 2001; Park et al. 2007; Huang et al. 2009). Therefore, we also tested whether nicotine (500 nM, equivalent to smoker plasma concentrations) (Ghosheh et al. 2001) can confer neuroprotection of cholinergic neurons. We found that when compared to NGF, neither nicotine nor bFGF had any apparent effect as measured by the number of VAChT-positive neurons and therefore excluded these agents in subsequent analyses (Online Resource 1a). Although the combination of NGF and BMP9 resulted in significantly enhanced cholinergic neuron survival at E14, there was less effect at E19 (Online Resource 1b). BMP9 also promotes glial overgrowth and fasciculation (Lopez-Coviella et al. 2005). Therefore, we opted to exclude BMP9 as well in our final culture protocol. A cocktail of all 3 growth factors (3XGF) performed no better than, or in some cases worse, than NGF alone, possibly due to glial overgrowth (Online Resource 1a and data not shown).

Under growth conditions with NGF supplementation, co-cultures could be maintained with a healthy morphology for greater than 3 weeks (Fig. 1d). The proportion of cholinergic neurons varied greatly however, depending on various conditions such as embryo age at dissection (E19 vs. E14) or ratio of septal:hippocampal cells (Fig. 1e, Online Resource 1a). We observed that in general, culturing neurons from E14 produced the highest percentage of cholinergic neurons (attaining up to ~7% of total cell population) over a wide range of septal to hippocampal cell ratios (Fig. 1e, Online Resource 1a). For E19 dissections however, NGF 3:1 was the only condition of those tested that grew well and approached the cholinergic survival rates of E14 cultures (Fig. 1e, Online Resource 1a). In the remainder of the study, we selected E19 3:1 NGF cultures for further characterization because E14 cultures are technically more demanding to prepare than E19.

Cholinergic neurons strongly expressed the cholinergic markers VAChT, choline acetyltransferase (ChAT), and the high-affinity choline transporter (ChT1) (Fig. 1f). All cholinergic neurons also expressed molecular machinery necessary for the release of GABA, including VGAT and GAD65 (Fig. 1g). The large majority of neurons that lacked VAChT expression were presumably glutamatergic or GABAergic (Fig. 1d, asterisk). VAChT+ neurons retained the magnocellular phenotype in culture (mean soma diameter 30 μm, range 19–42 μm). This size is strikingly similar to that reported for BFCNs in the intact brain (mean 30 μm, range 18–43 μm) (Woolf and Butcher 2011). VAChT-lacking PSD-93+ (non-cholinergic) neurons had significantly smaller soma size than cholinergic neurons (mean 20 μm, range 19–29 μm) (Fig. 1h), consistent with previous *in vivo* observations for hippocampal pyramidal neurons (range 15–30 μm) (Peacock et al. 1979; Johansson et al. 1992; Eslamizade et al. 2016). Thus, the co-cultured neurons bore molecular and morphological resemblance to *in vivo* counterparts.

### Development of mature cholinergic synapses and neurons *in vitro*

Cholinergic synapse formation has not been well documented for primary septo-hippocampal cultures grown over extended periods. Previous studies showed that septal neuron presynaptic terminals contact GABAergic synapses marked by the inhibitory scaffold protein gephyrin in the CA1 region of mouse hippocampus (Takács et al. 2018), but cholinergic receptors were not examined. In contrast, another study in peripheral ganglion neurons showed that cholinergic receptor subunits were highly associated with PSD-93, an excitatory scaffold protein (Parker et al. 2004), but CNS cholinergic neuron association with PSD-93 was not assessed. Thus, data are lacking that examine all three cholinergic components (presynapse, receptors, and scaffold) in central neurons.

**Fig. 2:**
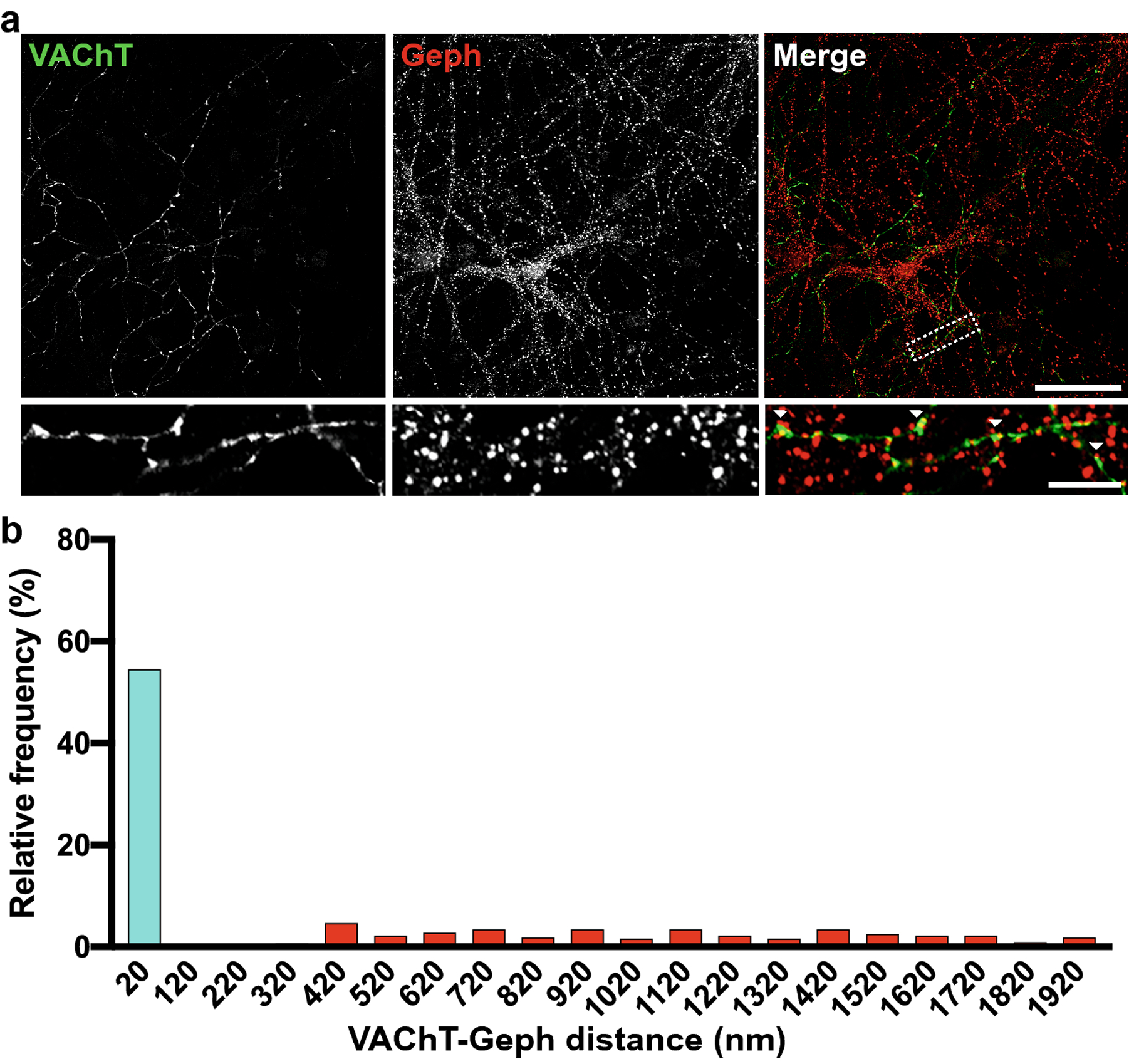
Spatial relationship between VAChT and gephyrin. (**a**) Immunocytochemistry of neurons from septal-hippocampal co-cultures (DIV 21) using guinea pig anti-VAChT (green) and rabbit anti-gephyrin (red) antibodies. Representative high magnification dendritic segments shown below are taken from white dotted region in upper image. (**b**) Frequency histogram of distances between VAChT puncta and nearest neighboring gephyrin puncta along innervated dendrites (n=30 segments from 3 coverslips and 3 independent cultures). Arrowheads indicate sites of overlapping puncta. Scale bars in low and high magnification images represent 25 μm and 5 μm, respectively. Bin size: 100 nm. Median/minimum/maximum: 0/0/3771 nm (maximum not shown in truncated graph). Mean ± SEM = 553.1 ± 41.6 nm.

To address this issue, we performed double labeling studies in our septal-hippocampal co-cultures, using VAChT antibodies to identify the cholinergic presynaptic component in combination with antibodies against different postsynaptic proteins. We observed that VAChT was highly colocalized and overlapped with the inhibitory scaffold gephyrin along innervated dendrites (Fig. 2a). The distance between each VAChT cluster and the nearest gephyrin puncta staining was measured, and we found that the majority of distances were in the 0–20 nm range (Fig. 2b). Such close apposition with evident overlap suggests that septal neurons were forming GABAergic synapses onto target cells, consistent with *in vivo* studies (Takács et al. 2018). However, a subpopulation of VAChT puncta were found to be distant from gephryin, and may represent terminals that did not form productive synapses *in vitro*.

**Fig. 3:**
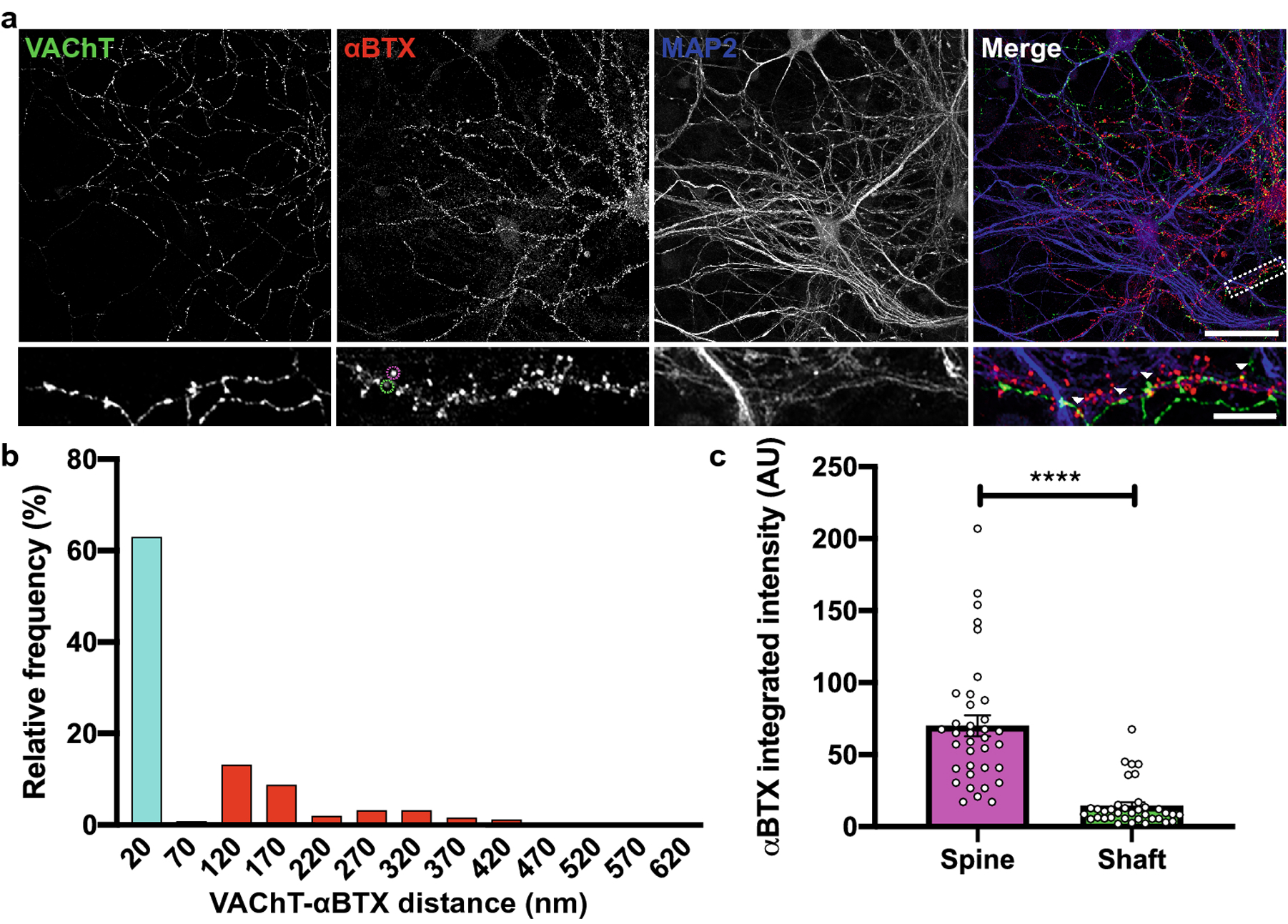
Spatial relationship between VAChT and α-bungarotoxin. (**a**) Immunocytochemistry of co-cultured primary septal-hippocampal neurons (DIV 21) using rabbit anti-VAChT (green), Alexa-555 conjugated α7 nAChR antagonist, α-bungarotoxin (α-BTX) (red), and chicken anti-microtubule-associated protein 2 (MAP2) (blue) antibodies. Representative high magnification dendritic segments shown below are taken from white dotted region in upper image. (**b**) Frequency histogram of distances between VAChT puncta and the nearest neighboring α-BTX-Alexa 555 puncta along innervated dendrites (n=30 segments from 3 coverslips and 3 independent cultures). Arrowheads indicate sites of close apposition/overlap. Scale bars in low and high magnification images represent 25 μm and 5 μm, respectively. Bin size: 100 nm. Median/minimum/maximum: 0/0/931 nm (maximum not shown in truncated graph). Mean ± SEM = 87.8 ± 9.5 nm. (**c**) Quantification of α-BTX-Alexa 555 integrated intensity on the dendritic spine and shaft (representative examples shown as dotted purple and green circles, respectively, in high magnification α-BTX image). ****p<0.0001, unpaired two-tailed Student’s t-test.

Next, we determined the spatial relationship between cholinergic neurons and α7 nicotinic acetylcholine receptors (α7 nAChRs), one of the most abundant ACh receptor subtypes in the hippocampus (Alkondon and Albuquerque 1993, 2004; Rubboli et al. 1994; Dani 2015). In order to visualize α7 nAChRs, we used the specific antagonist α-BTX conjugated to Alexa-555 to live-label our cultures (Fig. 3a). The specificity of α-BTX-Alexa-555 labeling for α7 nAChRs was verified by complete competition of signal with the highly selective α7 antagonist methyllycaconitine (MLA) (Online Resource 2a). Interestingly, α-BTX-Alexa 555 frequently labeled dendrites of VAChT-lacking neurons (Online Resource 2b), suggesting the expression of α7 nAChRs on cholinergic target cells of the hippocampus. However, in a subset of axons we could also detect precise labeling of VAChT+ terminals with α-BTX-Alexa-555, suggesting that α7 nAChRs is also found presynaptically on some septal afferents (Online Resource 2b).

We therefore triple-labeled our co-culture with VAChT, α-BTX-Alexa-555, and the neuronal dendrite marker microtubule associated protein 2 (MAP2) to restrict analysis of α7 nAChRs to septal contacts onto hippocampal target cells (Fig. 3a). The frequency histogram of the distances between VAChT and α-BTX puncta indicate that 63% of the puncta fall in the 0–20 nm bin, consistent with close apposition (Fig. 3b). To determine whether α7 nAChRs were found on dendritic spines vs. shafts, we quantified α-BTX-Alexa-555 mean integrated intensity and found that fluorescence signal was ~5-fold more enriched in dendritic spines compared to the shaft (Fig. 3c), suggesting that α7 nAChRs localize on spines and play a role in spine integrity as reported (Morley and Mervis 2013).

**Fig. 4:**
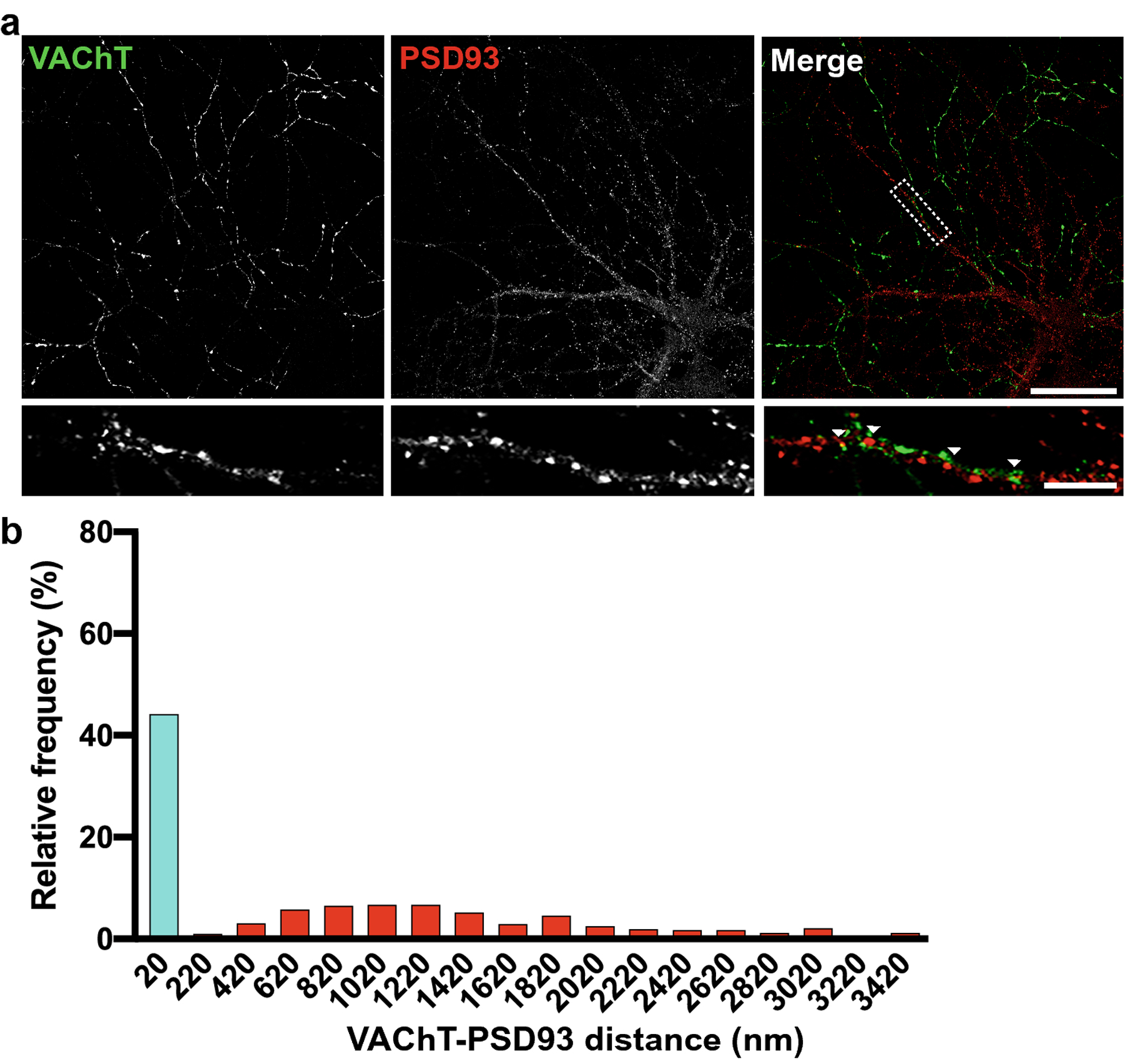
Spatial relationship between VAChT and PSD-93. (**a**) Immunocytochemistry of septal-hippocampal co-cultures (DIV 21) using rabbit anti-VAChT (green) and mouse anti-PSD-93 (red) antibodies. Representative high magnification dendritic segments shown below are taken from white dotted region in upper image. (**b**) Frequency histogram of distances between VAChT puncta and nearest neighboring PSD-93 puncta along innervated dendrites (n=30 segments from 3 coverslips and 3 independent cultures). Arrowheads indicate sites of juxtaposition. Scale bars in low and high magnification images represent 25 μm and 5 μm, respectively. Bin size: 100 nm. Median/minimum/maximum: 561/0/4219 nm (maximum not shown in truncated graph). Mean ± SEM = 819.3 ± 42.2 nm.

Lastly, we examined the spatial relationship between VAChT and PSD-93. PSD-93 colocalized nearly completely with the canonical glutamatergic scaffold protein PSD-95 and was used as a marker of excitatory synapses and postsynaptic densities (PSDs) (Online Resource 3) (Elias et al. 2006). Double immunolabeling of co-cultures demonstrated that 40% of VAChT clusters were found within 0–20 nm of PSD-93 (Fig. 4a,b), suggesting that these two proteins were also highly apposed.

**Fig. 5:**
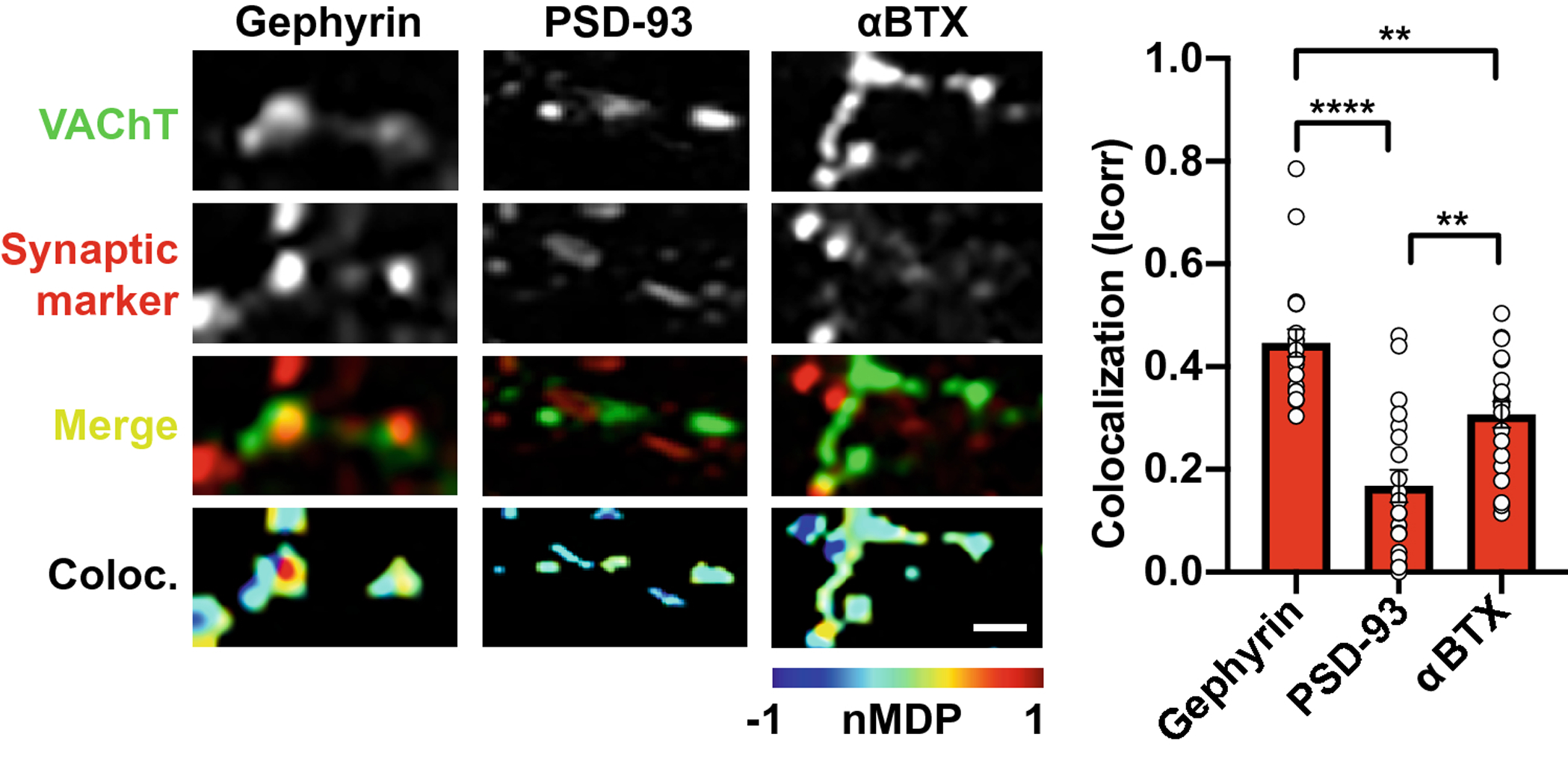
Colocalization of VAChT with gephyrin, PSD-93, and α-BTX. (*left*) Immunocytochemistry of cultured septal-hippocampal rat neurons (DIV 21) using VAChT (green) antibodies co-labeled with synaptic markers (red) as indicated above each image, with merge shown (yellow). Heat map at bottom depicts the degree of colocalization as normalized mean deviation product (nMDP) ranging between 0 and 1, representing no or perfect colocalization, respectively. Scale bar is 2 μm. (*right*) Quantification of the index of correlation (I_corr_) as a measure of colocalization is shown. VAChT is most colocalized with gephyrin (mean = 0.45), less colocalized with α-BTX (mean = 0.31), and least colocalized with PSD-93 (mean = 0.17). n= 15 segments from 3 coverslips and 3 independent cultures. **p=0.0025 (gephyrin vs. α-BTX), **p=0.0026 (PSD-93 vs. α-BTX), ****p<0.0001 (gephyrin vs. PSD-93); one-way ANOVA with multiple Tukey *post-hoc* test.

Upon closer examination of the immunostaining images, we observed that the precise distributions of the different postsynaptic markers relative to VAChT were not equivalent. For instance, VAChT appeared to be overlapping significantly with gephyrin, but lay more adjacent to PSD-93. To quantify these spatial relationships, we measured the degree of colocalization of VAChT with gephyrin, PSD-93, and α-BTX-Alexa-555 puncta in our septal-hippocampal co-cultures (Fig. 5). A heat map indicates the degree of colocalization expressed as normalized mean deviation product (nMDP), ranging between 0 (no colocalization) and 1 (perfect colocalization), and quantified as the index of correlation (I_corr_) (Fig. 5). We found that VAChT colocalization was relatively high with gephyrin (mean of 0.45), intermediate with α-BTX (mean of 0.31), and lowest with PSD-93 (mean of 0.17). These data, together with the interpuncta distance results, suggest that VAChT exists in close proximity to all of these postsynaptic factors, yet adopts distinct spatial organization with respect to inhibitory, excitatory, and cholinergic receptors.

## DISCUSSION

A vast literature comprising tens of thousands of citations has been generated using primary hippocampal cultured neurons, yielding a tremendous body of knowledge encompassing virtually all aspects of hippocampal neuronal development, structure, plasticity, and dysfunction. In stark contrast, primary septal-hippocampal co-cultured neurons have not been exploited nearly as extensively in the field. One reason may be the difficulty in maintaining such cultures for extended periods to allow for full developmental maturation. Additionally, while cultured striatal cholinergic interneurons have been characterized in detail (Schock et al. 2010), previous septal co-culture systems did not verify cholinergic neuron identity or synapse formation in a rigorous manner, and thus quality control has remained questionable.

Here, we have developed, validated, and described in detail an optimized septal-hippocampal co-culture system that features several improvements. The cultures are reproducible and robust, capable of long-term growth (over 3 weeks *in vitro*), which permits development to approximate a mature state. Although E14 cultures were slightly superior to E19, we chose the latter as the brains are larger and dissections are more straightforward to perform, whereas the more difficult E14 dissections would likely limit utilization and adoption of this methodology. Since E19 is the most widely used embryonic culture date, our procedure can also be easily incorporated with existing protocols. Moreover, E19 hippocampal-only cultures are well characterized and findings can be more directly compared with septal-hippocampal co-cultures obtained from the same dissection date.

**Fig. 6:**
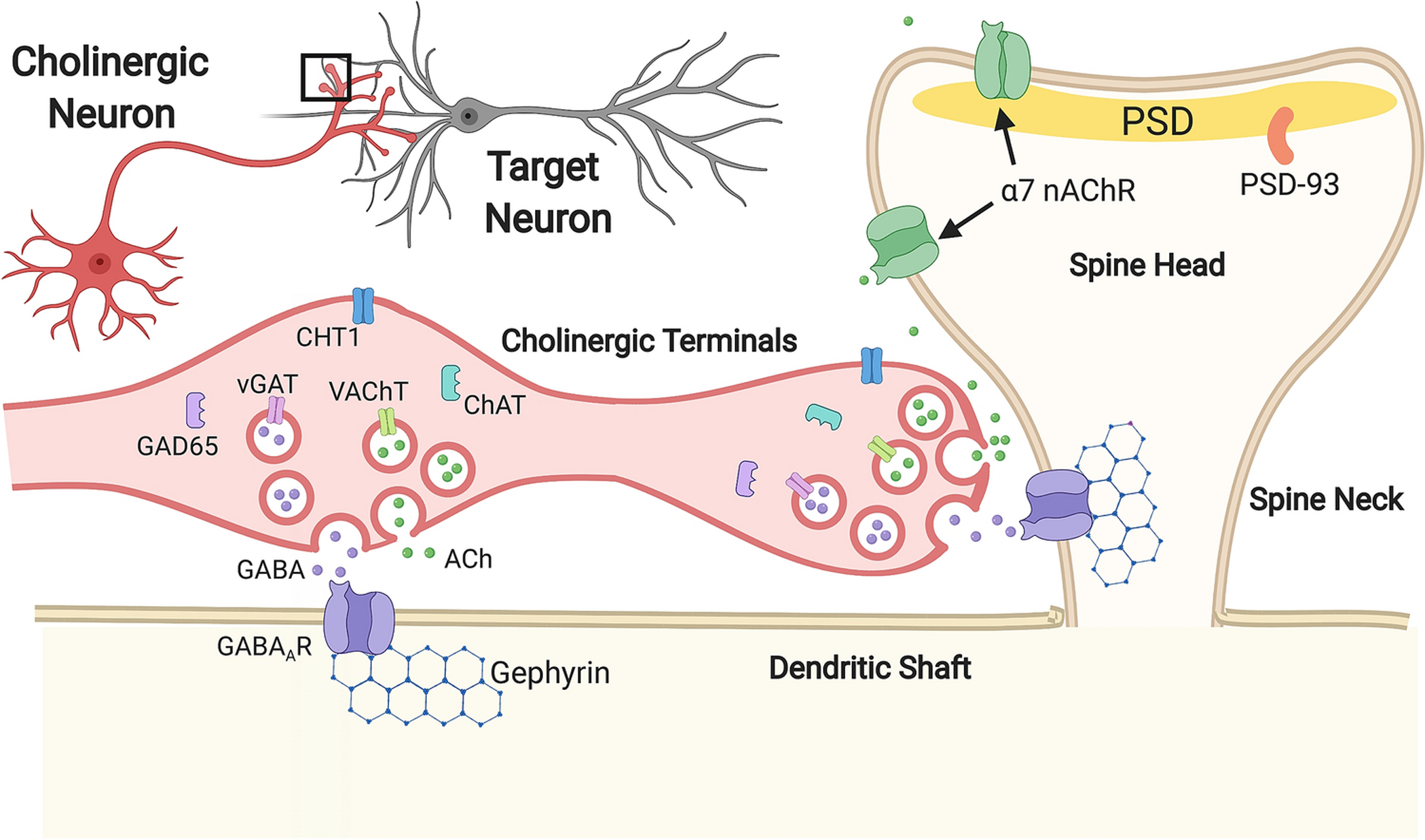
Schematic of septal-hippocampal co-cultured neurons and synapses. Cholinergic neurons in mature septal-hippocampal co-cultures express molecular machinery necessary for the release of ACh and GABA, including vesicular ACh transporter (VAChT), choline acetyltransferase (ChAT), and high affinity choline transporter (CHT1). Cholinergic terminals also express the 65 kDa isoform of glutamic acid decarboxylase (GAD65) and the vesicular GABA transporter (VGAT), machinery necessary for GABA release. Cholinergic terminals form GABAergic synapses on the dendritic shaft and dendritic spine neck marked by the inhibitory scaffold protein gephryin. PSD-93 is enriched at PSDs and is more distant from VAChT, suggesting a nonsynaptic localization adjacent to terminals. α7 nAChRs are present throughout the dendritic spine head but with intermediate colocalization with VAChT, suggesting an extrasynaptic/perisynaptic localization of α7 with respect to the PSD.

The cholinergic and hippocampal target neurons retain distinct identities as demonstrated morphologically by soma size as well as by cell type-specific markers. Furthermore, the concentrated presence of α7 nAChRs in dendritic spines is consistent with previous studies conducted in the rodent hippocampal brain slice with preserved cholinergic terminals (Fabian-Fine et al. 2001). We used VAChT as the main cholinergic marker instead of AChE, since VAChT is a more specific marker for cholinergic neurons. Importantly, this is the first report to our knowledge to visualize central mammalian cholinergic synapses *in vitro*. The resulting synapses occur between physiological partners and resemble *in vivo* counterparts in terms of synaptic protein composition and localization. Notably, a previous study showed that BFCN terminals form synapses onto GABAergic postsynaptic sites marked by the inhibitory scaffold protein gephryin, indicating co-transmission of GABA and ACh (Takács et al. 2018). In our cultures, VAChT+ terminals that innervated target dendritic segments were also extensively colocalized with gephryin, suggestive of inhibitory synapses formed by septal boutons on dendritic shafts (see schematic in Fig. 6). Moreover, we verified that cholinergic neurons contained the presynaptic machinery necessary for GABA release, including VGAT and GAD65.

Additionally, we extended the findings of Takács et al. further by examining the association of VAChT+ terminals with cholinergic receptors and excitatory postsynaptic sites, which, to our knowledge, has not been performed previously for central cholinergic synapses. We observed that VAChT puncta were found in very close proximity with both α7 nAChRs and PSD-93. However, the degree of colocalization for VAChT with α7 nAChRs was significantly less than with gephyrin, and even less for PSD-93. Although the resolution of light microscopy does not permit absolutely precise localization of these proteins, the low overlap between VAChT with PSD-93 suggests that VAChT may be adjacent to the PSD where PSD-93 is enriched. These results are consistent with previous observations using electron microscopy that septal terminals can also form synapses onto dendritic spine necks *in vivo* (Takács et al. 2018). The intermediate level of overlap we observed between VAChT and α-BTX suggests that α7 nAChRs may be extrasynaptic / perisynaptic with respect to the PSD in the dendritic spine head, potentially allowing for volume transmission from nearby cholinergic terminals (Fig. 6). Thus, these co-cultures recapitulate several key aspects of *in vivo* BFCN development, structure, and connectivity.

In this body of work, we did not examine for the presence of presynaptic cholinergic receptors on hippocampal glutamatergic terminals. The presence and role of presynaptic cholinergic receptors in glutamatergic terminals has been previously examined in the intact rodent slice (Gray et al. 1996; Fabian-Fine et al. 2001; Sharma and Vijayaraghavan 2003; Cheng and Yakel 2014). For instance, compelling evidence suggests that presynaptic α7 nAChRs on mossy fiber terminals enhance glutamatergic transmission (Cheng and Yakel 2014). Here, our focus was to identify and characterize both sides of the central cholinergic synapse, which has not been reported previously. Therefore, we used VAChT and αBTX-555 as pre- and post-synaptic cholinergic markers, respectively. We acknowledge that given the aforementioned reports in the literature, αBTX-555 may indeed be localized on presynaptic glutamatergic terminals. However, in line with previous studies (Fabian-Fine et al. 2001), the morphology of the αBTX-555 staining used in our colocalization analysis may reflect a postsynaptic localization on a dendritic spine. Higher resolution microscopy methods will be needed to unambiguously distinguish between pre-and postsynaptic cholinergic receptor distribution at glutamatergic synapses.

The availability of a robust septal-hippocampal co-culture system will readily allow further investigation into the molecular composition and localization of cholinergic synapses, particularly when combined with super-resolution microscopy techniques. *In vitro* cultures also allow rapid analysis of various stimulation paradigms that should advance knowledge of different forms of cholinergic synaptic plasticity. Significantly, given that recent studies show that loss of BFCNs in AD precedes and predicts the degeneration observed in the entorhinal cortex (Schmitz et al. 2016), this system will shed light onto the mechanisms of mature cholinergic neuron and synapse vulnerability in disease.

## Supplementary Material

10571_2020_948_MOESM1_ESM

## LIST OF ABBREVIATIONS

αBTX: α-Bungarotoxin
AChE: Acetylcholinesterase
AD: Alzheimer’s Disease
BFCN: Basal forebrain cholinergic neurons
bFGF: Basic fibroblast growth factor
BMP9: Bone morphogenetic protein 9
ChAT: Choline acetyltransferase
CHT1: High-affinity choline transporter
CNS: Central nervous system
DIV: Day in vitro
GABA: Gamma-aminobutyric acid
GAD65: Glutamate decarboxylase 65
Geph: Gephyrin
MAP2: Microtubule associated protein 2
MLA: Methyllycaconitine
nAChR: Nicotinic acetylcholine receptor
NGF: Nerve growth factor
nMDP: Normalized mean deviation product
PSD-93: Postsynaptic density 93
PSD-95: Postsynaptic density 95
VAChT: Vesicular acetylcholine transporter
VGAT: Vesicular GABA transporte
