# Supplementary material for: Central cholinergic synapse formation in optimized primary septal-hippocampal co-cultures
Source: Cell Mol Neurobiol. Author manuscript; Available in PMC 2022 Nov 1. (PMC7914286; doi:10.1007/s10571-020-00948-6)
Supplement: 10571_2020_948_MOESM1_ESM [file NIHMS1624718-supplement-10571_2020_948_MOESM1_ESM.pdf]

Central cholinergic synapse formation in optimized primary septal-hippocampal co-cultures

Sarra Djemil[1], Claire R. Ressel[2], Mai Abdel-Ghani[1], Amanda K. Schneeweis[1], and Daniel T.S. Pak[1,3]*

[1]Department of Pharmacology and Physiology, Georgetown University Medical Center, Washington, District of Columbia, USA

[2] Department of Biology, Georgetown University, Washington, District of Columbia, USA

[3]Interdisciplinary Program in Neuroscience, Georgetown University Medical Center, Washington, District of Columbia, USA

ORCiDs: D.T.S.P: 0000-0001-5930-2589; S.D.: 0000-0001-8622-650X; C.R.R: 0000-0003-4361-104X; M.A.G: 0000-0002-9061-1045; A.K.S.: 0000-0003-4141-6064

*To whom correspondence should be addressed.

Target	Species	Dilution	Source /Cat#/RRID	Specificity/Validation
Vesicular Acetylcholine Transporter (VAChT)	Rabbit	1:500	(Synaptic Systems Cat# 139 103, RRID: AB_887864)	**KO verified** (manufacturer's information)<br>**No staining in purely hippocampal cultures** which are devoid of cholinergic cells (our data)
VAChT	Guinea-pig	1:500	(Synaptic Systems Cat# 139 105, RRID: AB_10893979)	**Gives identical staining to the K.O. verified 139 103 rabbit-anti-vAChT antibody** (our data)
Gephyrin	Rabbit	1:500	(Synaptic Systems Cat# 147 008, RRID: AB_2619834)	**KO verified** (manufacturer's information)
Postsynaptic density 93 (PSD93)/ Chapsyn-110	Mouse	1:250	(UC Davis/NIH NeuroMab Cat# 75-284, RRID: AB_11001825)	**KO verified** (manufacturer's information)
Postsynaptic density 95 (PSD-95)	Rabbit	1:200	(Cell Signaling Technology Cat# 3450, RRID: AB_2292883)	**Increased network activity decreases PSD95 staining** (our data, (Lee et al. 2017))
High affinity choline transporter (CHT1)	Mouse	1:500	(Synaptic Systems, cat. # 216 011, RRID: AB_2301977)	**KO verified** (manufacturer's information)<br>**Colocalizes with VAChT** (our data)<br>**No staining in purely hippocampal cultures** which are devoid of cholinergic cells (our data)
Choline acetyltransferase (ChAT)	Goat	1:400	(Millipore, cat. # AB144p, AB_2079751)	**VAChT** (our data)<br>**No staining in purely hippocampal cultures** which are devoid of cholinergic cells (our data)
Vesicular GABA transporter (VGAT)	Mouse	1:500	(Synaptic Systems, cat. # 131 011, RRID: AB_887872)	**KO verified** (manufacturer's information)
Glutamate decarboxylase 65 (GAD65)	Rabbit	1:250	(Millipore, cat. # AB5082, RRID: AB_2107925)	**Colocalizes with VGAT** (our data)
α-Bungarotoxin Alexa Fluor 555 conjugate	NA	1 μg/mL	(Thermo Fisher Scientific Cat# B35451, RRID: AB_2617152)	**MLA causes complete competition of α-Bungarotoxin Alexa Fluor 555 positive staining** (our data)

Online Resource Table 1 Validation of antibodies and α-bungarotoxin

Software		
Adobe Illustrator	Adobe	https://www.adobe.com/creativecloud.html
Adobe Acrobat	Adobe	https://www.adobe.com/creativecloud.html
Colocalization Colormap plugin	Open source	https://sites.google.com/site/colocalizationcolormap/installationanduse
Fiji ImageJ software	Open source	https://fiji.sc/
GraphPad PRISM	Graphpad	https://www.graphpad.com/scientific-software/prism/
Leica Application Suite X (LAS X)	Leica Microsystems	https://www.leica-microsystems.com/products/microscope-software/p/leica-las-x-ls/

Online Resource Table 2 Software used for data acquisition and analysis

a

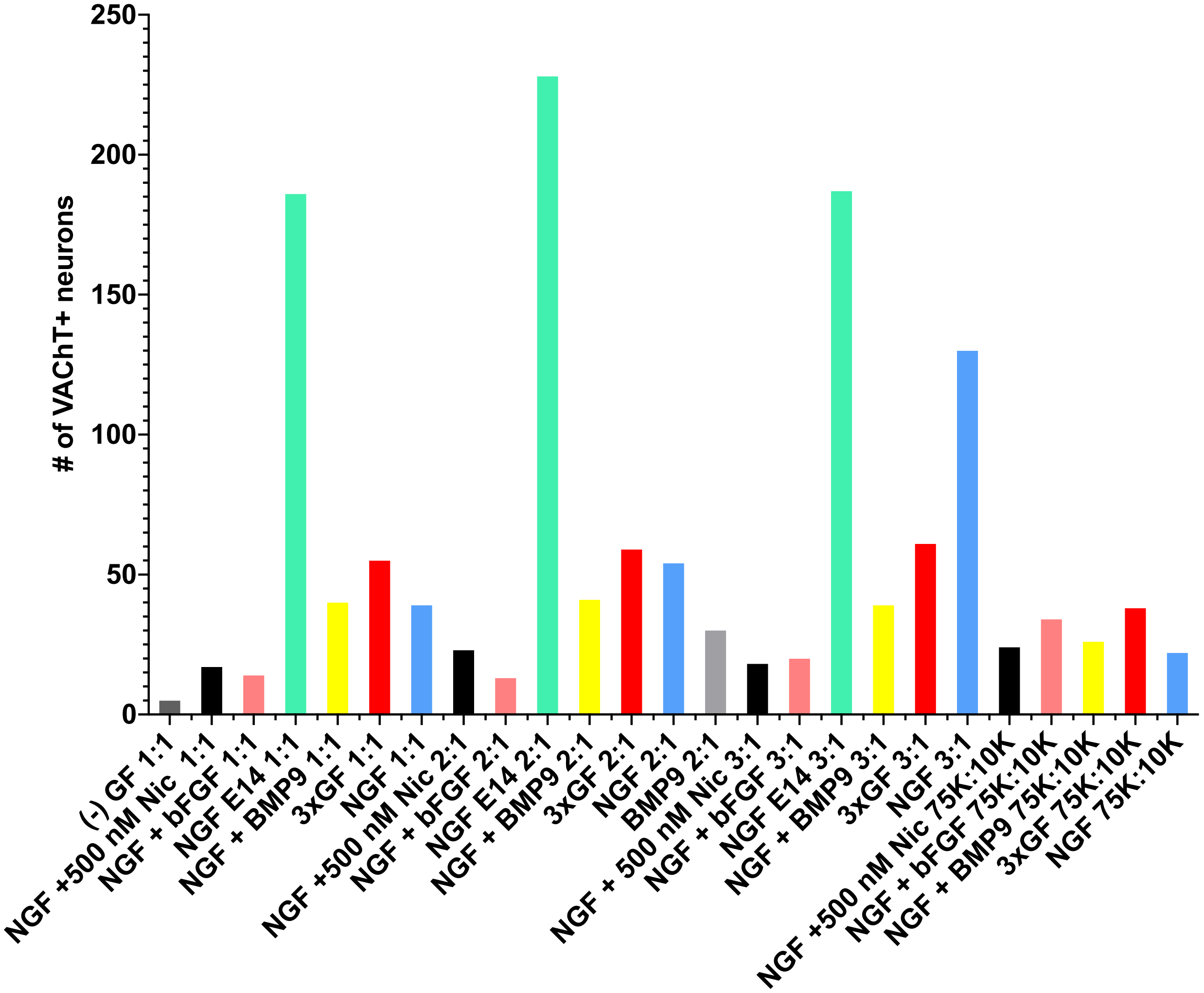

# of VAChT+ neurons
250
200
150
100
50
0
(-) GF 1:1
NGF +500 nM Nic 1:1
NGF + bFGF 1:1
NGF E14 1:1
NGF + BMP9 1:1
3xGF 1:1
NGF 1:1
NGF +500 nM Nic 2:1
NGF + bFGF 2:1
NGF E14 2:1
NGF + BMP9 2:1
3xGF 2:1
NGF 2:1
BMP9 2:1
NGF + 500 nM Nic 3:1
NGF + bFGF 3:1
NGF E14 3:1
NGF + BMP9 3:1
3xGF 3:1
NGF 3:1
NGF +500 nM Nic 75K:10K
NGF + bFGF 75K:10K
NGF + BMP9 75K:10K
3xGF 75K:10K
NGF 75K:10K

b

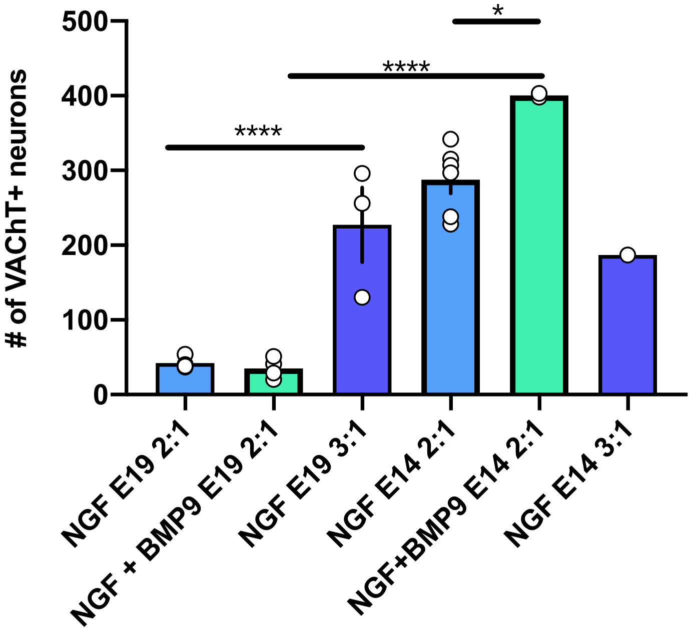

# of VAChT+ neurons
500
400
300
200
100
0
*
****
****
NGF E19 2:1
NGF + BMP9 E19 2:1
NGF E19 3:1
NGF E14 2:1
NGF+BMP9 E14 2:1
NGF E14 3:1

Online Resource 1

Growth conditions screened. (**a**) Various growth factors and conditions were chosen based on previously published works (Hartikka and Hefti 1988; Ryan et al. 2001; Pak et al. 2001; Lopez-Coviella et al. 2005; Park et al. 2007; Schnitzler et al. 2008; Huang et al. 2009; Schock et al. 2010; Haam et al. 2018) to test for support of cholinergic phenotypes. Number of VAChT+ neurons were counted from a single culture (n=1 coverslip). (**b**) Additional quantification of number of VAChT+ neurons. NGF+BMP9 E14 2:1 yielded the most VAChT+ neurons of the conditions tested. NGF+BMP9 E14 2:1 (n=2 coverslips from a single culture), NGF+BMP9 E19 2:1 (n=4 coverslips from 3 independent cultures), NGF E19 3:1 (n=3 coverslips from 3 independent cultures), NGF E19 2:1 (n=4 coverslips from 3 independent cultures), NGF+BMP9 E14 2:1 (n= 2 coverslips from a single culture), NGF E14 2:1 (n=6 coverslips from 2 independent cultures). ****p<0.0001, *p=0.0437. One-way ANOVA followed by Holm-Sidak's multiple comparison test. All conditions are E19 unless otherwise indicated as E14.

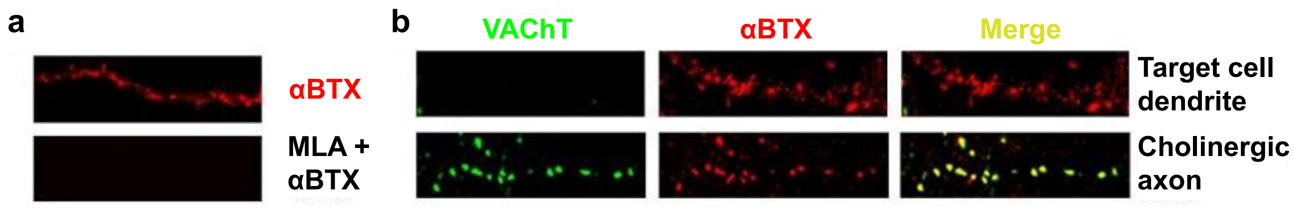

Online Resource 2

Detection of α7 nAChRs. (**a**) Labeling of α7 in septal-hippocampal co-cultures using α-bungarotoxin (α-BTX) conjugated to Alexa-555. Signal from α-BTX is completely abolished by pre-incubation with 10 μM MLA (α7 nAChR selective competitive antagonist) for 15 minutes. (**b**) Top, α-BTX-Alexa-555 labels dendritic spines of non-cholinergic cells. Bottom, α-BTX-Alexa-555 precisely labels a subset of VAChT+ terminals indicating α7 enrichment in some, but not all, BFCN presynaptic terminals. Scale bar is 5 μm.

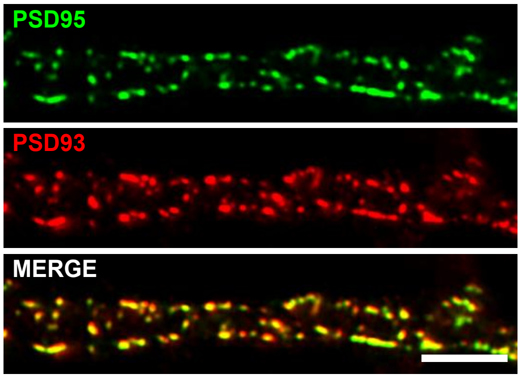

Online Resource 3

Colocalization of PSD-93 and PSD-95. Immunocytochemistry of septal-hippocampal co-cultured neurons (DIV 21) from Sprague Dawley embryonic rats using anti-PSD-95 (green) and anti-PSD-93 (red) antibodies. Representative high magnification view of dendritic segments. Scale bar is 5 μm.

REFERENCES

Haam J, Zhou J, Cui G, Yakel JL (2018) Septal cholinergic neurons gate hippocampal output to entorhinal cortex via oriens lacunosum moleculare interneurons. Proc Natl Acad Sci U S A 115:E1886–E1895. https://doi.org/10.1073/pnas.1712538115

Hartikka J, Hefti F (1988) Development of septal cholinergic neurons in culture: plating density and glial cells modulate effects of NGF on survival, fiber growth, and expression of transmitter-specific enzymes. J.neurosci 8:2967–2985

Huang LZ, Parameswaran N, Bordia T, et al (2009) Nicotine is neuroprotective when administered before but not after nigrostriatal damage in rats and monkeys. J Neurochem 109:826–837. https://doi.org/10.1111/j.1471-4159.2009.06011.x

Lee Y, Lee JS, Lee KJ, et al (2017) Polo-like kinase 2 phosphorylation of amyloid precursor protein regulates activity-dependent amyloidogenic processing. Neuropharmacology 117:387–400. https://doi.org/10.1016/j.neuropharm.2017.02.027

Lopez-Coviella I, Follettie MT, Mellott TJ, et al (2005) Bone morphogenetic protein 9 induces the transcriptome of basal forebrain cholinergic neurons. Proc Natl Acad Sci U S A 102:6984–6989. https://doi.org/10.1073/pnas.0502097102

Pak DT, Yang S, Rudolph-Correia S, et al (2001) Regulation of dendritic spine morphology by SPAR, a PSD-95-associated RapGAP. Neuron 31:289–303

Park HJ, Lee PH, Ahn YW, et al (2007) Neuroprotective effect of nicotine on dopaminergic neurons by anti-inflammatory action. Eur J Neurosci 26:79–89. https://doi.org/10.1111/j.1460-9568.2007.05636.x

Ryan RE, Ross SA, Drago J, Loiacono RE (2001) Dose-related neuroprotective effects of chronic nicotine in 6-hydroxydopamine treated rats, and loss of neuroprotection in α4 nicotinic

receptor subunit knockout mice. Br J Pharmacol 132:1650–1656

Schnitzler AC, Lopez-Coviella I, Blusztajn JK (2008) Purification and culture of nerve growth factor receptor (p75)-expressing basal forebrain cholinergic neurons. Nat Protoc 3:34–40

Schock SC, Jolin-Dahel KS, Schock PC, et al (2010) Striatal interneurons in dissociated cell culture. Histochem Cell Biol 134:1–12. https://doi.org/10.1007/s00418-010-0707-9
